# Low-Power Graphene/ZnO Schottky UV Photodiodes with Enhanced Lateral Schottky Barrier Homogeneity

**DOI:** 10.3390/nano9050799

**Published:** 2019-05-24

**Authors:** Youngmin Lee, Deuk Young Kim, Sejoon Lee

**Affiliations:** 1 Quantum-Functional Semiconductor Research Center, Dongguk University—Seoul, Seoul 04623, Korea; ymlee@dongguk.edu (Y.L.); dykim@dongguk.edu (D.Y.K.); 2 Department of Semiconductor Science, Dongguk University—Seoul, Seoul 04623, Korea

**Keywords:** graphene, zinc oxide, Schottky photodiode, Schottky barrier homogeneity

## Abstract

The low-power, high-performance graphene/ZnO Schottky photodiodes were demonstrated through the direct sputter-growth of ZnO onto the thermally-cleaned graphene/SiO_2_/Si substrate at room temperature. Prior to the growth of ZnO, a thermal treatment of the graphene surface was performed at 280 °C for 10 min in a vacuum to desorb chemical residues that may serve as trap sites at the interface between graphene and ZnO. The device clearly showed a rectifying behavior with the Schottky barrier of ≈0.61 eV and an ideality factor of 1.16. Under UV illumination, the device exhibited the excellent photoresponse characteristics in both forward and reverse bias regions. When illuminating UV light with the optical power density of 0.62 mW/cm^2^, the device revealed a high on/off current ratio of >10^3^ even at a low bias voltage of 0.1 V. For the transient characteristics upon switching of UV light pulses, the device represented a fast and stable photoresponse (i.e., rise time: 0.16 s, decay time: 0.19 s). From the temperature-dependent current–voltage characteristics, such an outstanding photoresponse characteristic was found to arise from the enhanced Schottky barrier homogeneity via the thermal treatment of the graphene surface. The results suggest that the ZnO/graphene Schottky diode holds promise for the application in high-performance low-power UV photodetectors.

## 1. Introduction

Graphene-based hybrid and heterostructures with other inorganic and/or organic materials render more fascinating functionalities compared to conventional electronic and optoelectronic devices [1,2,3]. Among various graphene-based hetero-architectures, the graphene/inorganic semiconductor contacts have attracted much attention because of their ample potential for high-performance Schottky photodiodes (PDs) [4]. For example, the enhanced photoresponse characteristics with high sensitivity and fast response were demonstrated on various graphene-based Schottky PDs that were composed of typical inorganic semiconductor materials (e.g., Si [5,6], Ge [7], GaAs [8,9], CdSe [10], ZnO [11,12,13,14,15,16,17,18,19,20,21], etc.). Among them, ZnO is one of the most prospective materials for high-performance ultraviolet (UV) PDs because of its wide band gap and excitonic properties [22,23]. Due to the *c*-axis preference of wurtzite ZnO, moreover, ZnO thin films [18,19] or ZnO nanorods [20] can be easily grown on the defective graphene sheet that had been grown using chemical vapor deposition (CVD). Namely, the presence of C–O nucleation sites on CVD graphene allows us to fabricate a simple device scheme of the graphene/ZnO Schottky PD through the direct growth of ZnO on graphene. Furthermore, since the work function of CVD graphene could be controlled by thermal [24,25] and chemical [26] treatments, one can easily manipulate the Schottky barrier height at the graphene/ZnO interface. Owing to such advantages, very recently, high-performance graphene/ZnO Schottky UV PDs have been realized through various techniques; for example, direct growth of ZnO onto graphene by using CVD [16], chemical bath deposition [11], radio frequency (r.f.) magnetron sputtering [18,19], hydrothermal methods [20], and dispersion of ZnO nanorods onto graphene [12,13,14]. When using graphene as an active layer of the electronic and the optoelectronic devices, there are critical issues from chemical adsorbates and residues that might remain on the graphene surface during the graphene transfer step [24]. For instance, during the growth of ZnO on graphene, the chemical adsorbates and residues will degrade the lateral homogeneity of the Schottky barrier because they act as unnecessary dopants and/or contaminants at the graphene/ZnO interface. In Schottky PDs, the inhomogeneous barrier underneath the photon collection area may degrade the ideality factor (i.e., transport characteristics) [4], and will eventually restrict the photoresponse characteristics of the Schottky PDs. To realize the high-performance graphene/ZnO Schottky UV PDs, therefore, enhancing the lateral Schottky barrier homogeneity is essential. In other words, the chemical adsorbates and residues should be effectively removed prior to the growth of ZnO onto the graphene surface.

In light of all the above, we have investigated the enhancement of the lateral Schottky barrier homogeneity in the graphene/ZnO Schottky PDs. The devices were fabricated by direct sputtering of ZnO onto thermally-cleaned single-layer graphene (SLG). Namely, to improve the ZnO/SLG interface properties, in situ thermal cleaning of CVD SLG was performed just prior to the sputtering of ZnO in a single chamber. This simple method uses neither chemical nor physical treatments that may provide additional adsorption of gas molecules during subsequent handling of the sample in air ambience. Despite such advantages, according to our best survey, no previous works have reported on the in situ thermal treatment for fabricating a high-performance SLG/ZnO Schottky PD. Through the temperature-dependent electrical characterization, we analyzed the Schottky barrier homogeneity of the fabricated ZnO/SLG Schottky PDs. In addition, we thoroughly assessed the photoresponse characteristics of the devices due to varying UV powers.

## 2. Experimental Section

### 2.1. Preparation of SLG/SiO_2_/Si Substrate

**Figure 1:**
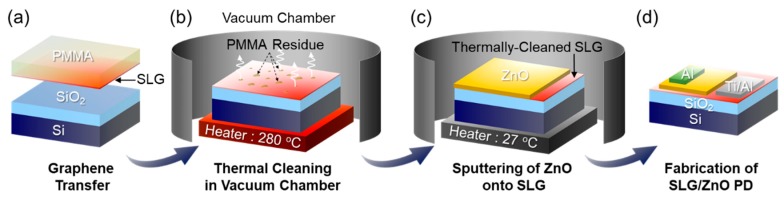
Schematic illustration for the fabrication of the ZnO/SLG Schottky PD: (**a**) SLG transfer step, (**b**) in situ thermal cleaning of SLG in vacuum sputtering chamber, (**c**) sputtering deposition of ZnO onto thermally-cleaned SLG, and (**d**) fabrication of the ZnO/SLG Schottky PD via lithographic techniques.

Figure 1 schematically illustrates the device fabrication procedures for the SLG/ZnO Schottky PD. First, the SLG sheet was grown on Cu foil using CVD and transferred onto the SiO_2_/Si substrate by using a poly(methyl methacrylate) (PMMA) transfer method (Figure 1a). Next, the sample was mounted in the sputtering chamber and was thermally cleaned at 280 °C for 10 min in a high vacuum (≈10^−6^ Torr) to eliminate chemical residues and/or molecular oxygens that might be adsorbed onto the SLG surface during the transfer process (Figure 1b).

### 2.2. Fabrication of ZnO/SLG Schottky PDs

The ZnO/SLG Schottky contacts were formed via the direct growth of the 200-nm-thick ZnO layer onto the SLG/SiO_2_/Si substrate through r.f. magnetron sputtering (Figure 1c). The sputtering process was performed at room temperature in Ar plasma ambient under the following conditions: Ar gas flow rate = 30 sccm, working pressure = 30 mTorr, and r.f. power = 150 W. After the growth of the ZnO layer onto SLG, the active area (*w* ≈ 10 μm, *l* ≈ 30 μm) was defined by conventional photolithography techniques. Finally, the Al (*t_Al_* ≈ 100 nm) and Ti/Al (*t_Ti_* ≈ 10, *t_Al_* ≈ 100 nm) ohmic electrodes for ZnO and SLG layer were formed using electron-beam evaporation and standard lift-off processes (Figure 1d). We here note that two different types of ZnO/SLG Schottky PDs were prepared by using as-transferred SLG and thermally-cleaned SLG so as to examine the effects of thermal cleaning on the device characteristics. For convenience, we refer the former and the later as a ZnO/AT-SLG Schottky PD and a ZnO/TC-SLG Schottky PD, respectively.

### 2.3. Measurements of Material and Device Characteristics

The Raman scattering characteristics of the as-transferred and thermally-cleaned SLG samples were measured by using a Renishaw Micro Raman spectrometer (Renishaw, Wotton-under-Edge, UK) under green laser excitation (λ = 514 nm). The topographic cross-section and the crystal structure of the ZnO layer were monitored through scanning electron microscopy (SEM) using an FE SEM XL-30 system (Phillips, Eindhoven, The Netherlands) and X-ray diffractometry (XRD) using a Bede D3 system (Bede Scientific Instruments Ltd., Durham, UK), respectively. The temperature-dependent electrical characteristics of the ZnO/SLG Schottky PDs were assessed at 300–400 K by using a Keysight B1500A semiconductor device parameter analyzer (Keysight Technologies, Santa Rosa, CA, USA). The photoresponse characteristics of the PDs were examined under light illumination using a 365-nm UV light emitting diode, wherein the UV power density (P_UV_) was varied from 0–0.77 mW/cm^2^ during photoresponse measurements.

## 3. Results and Discussion

**Figure 2:**
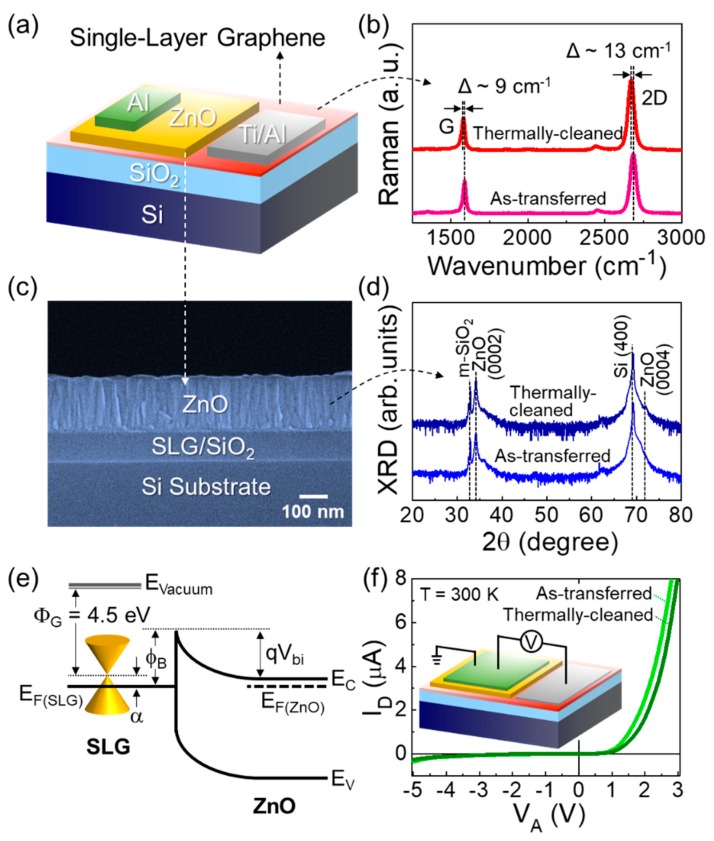
(**a**) Schematic of the ZnO/SLG Schottky PD. (**b**) Raman spectra of the as-transferred and the thermally-cleaned SLG sheets. (**c**) Cross-sectional view of the SEM image for the ZnO layer grown on the SLG/SiO_2_/Si substrate. (**d**) XRD patterns of the ZnO layers grown on the as-transferred and the thermally-cleaned SLG sheets. (**e**) Expected energy band diagram of the ZnO/SLG Schottky PD at thermal equilibrium. (**f**) I–V characteristics of the ZnO/AT-SLG and the ZnO/TC-SLG Schottky PDs. The inset of (f) shows the schematic configuration of the bias setup for the electrical characterization of the fabricated device.

Figure 2a schematically illustrates the final form of the fabricated ZnO/SLG Schottky PD, and Figure 2b shows the Raman spectra of the SLG sheets used for the device fabrication. Both the as-transferred and the thermally-cleaned SLG sheets revealed two predominant Raman features of G and 2D bands from high-quality graphene. For as-transferred SLG, the G and 2D peaks appeared at ≈1588 and 2684 cm^−1^, respectively, and the observed positions were blue-shifted from pristine graphene (i.e., G_pristine_ ≈ 1580–1585 cm^−1^, 2D_pristine_ ≈ 2635–2645 cm^−1^) [25]. This implies that our as-transferred SLG was doped by acceptor impurities from oxygen molecules [27] and/or chemical residues [28,29] adsorbed during the graphene transfer step. After thermal cleaning at 280 °C, both G and 2D peaks were shifted by 9 and 13 cm^−1^ toward their pristine graphene positions, respectively. Such a red-shift of both the G and 2D peaks depicts the decrease in charge trapping effects because the unintentional acceptors were effectively removed via thermal annealing at 280 °C [24,25]. Furthermore, no appearance of the defect-mediated D band at ≈1350 cm^−1^ indicates that vacuum-annealing at 280 °C caused no damage to the sp^2^ carbon bonds in SLG.

Onto the surface of high quality SLG, we deposited a 200-nm-thick ZnO layer at room temperature by using an r.f. magnetron sputtering technique. As shown in Figure 2c, the cross-sectional SEM image shows that the ZnO layer was effectively grown on SLG with a well-merged *c*-axis preferential columnar structure. The ZnO layers grown on the as-transferred and the thermally-cleaned SLG sheets exhibited a typical XRD pattern with the (000*l*) lattice phase (Figure 2d), which is indicative of the wurtzite structure of typical ZnO. In such a ZnO/SLG structure, the Schottky barrier (*ϕ*_B_ = Φ_SLG_ − χ_ZnO_) would be formed at the interface between SLG and ZnO (Figure 2e) because the work function of CVD SLG (Φ_SLG_ > ≈4.5 + *α* eV) [30] is greater than electron affinity of ZnO (χ_ZnO_ ≈ 4.1 eV) [31]. Here, the magnitude of *α* depends on the difference between the Dirac point and the Fermi level in CVD SLG, and it could be caused by the p-type doping effect from oxygen molecules and/or chemical residues. Thanks to the formation of the Schottky barrier, the SLG/ZnO Schottky PDs show a good rectifying behavior (Figure 2f).

**Figure 3:**
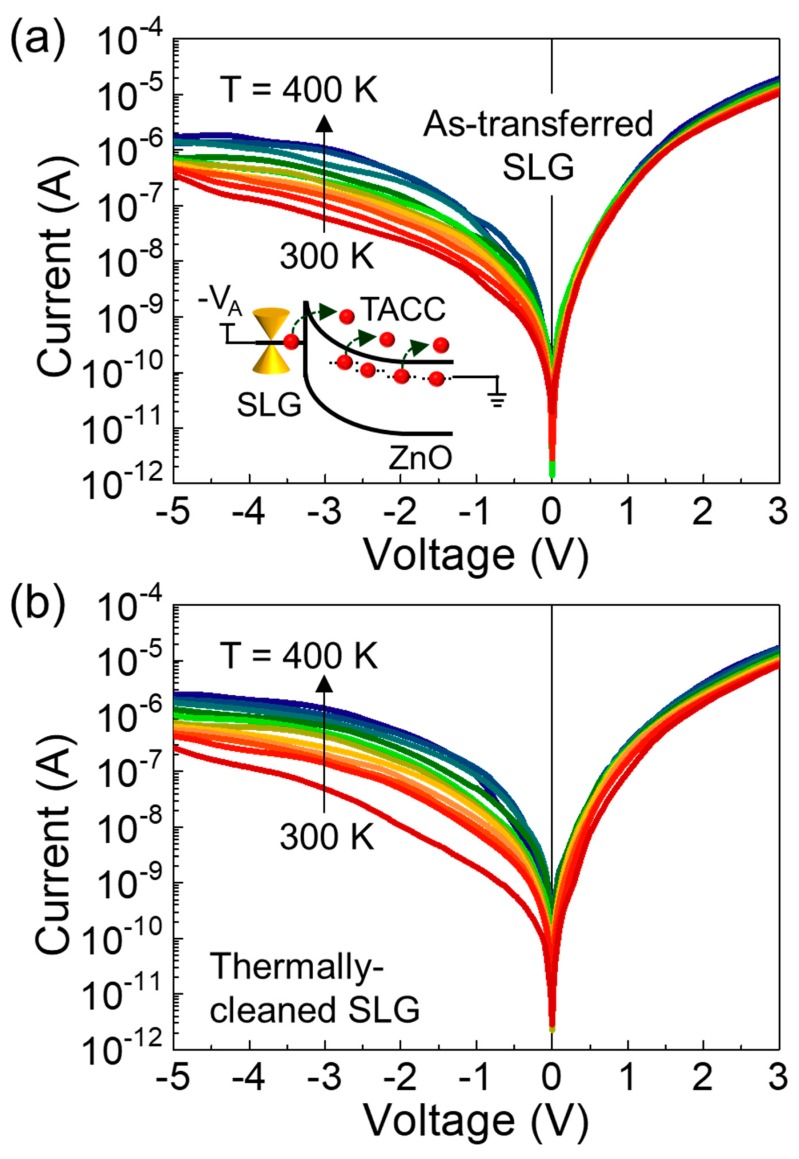
Temperature dependent I–V characteristics at 300–400 K of the (**a**) ZnO/AT-SLG and the (**b**) ZnO/TC-SLG Schottky PDs. The inset of (a) represents a contribution of TACC under the reverse bias state at the elevated temperature.

**Figure 4:**
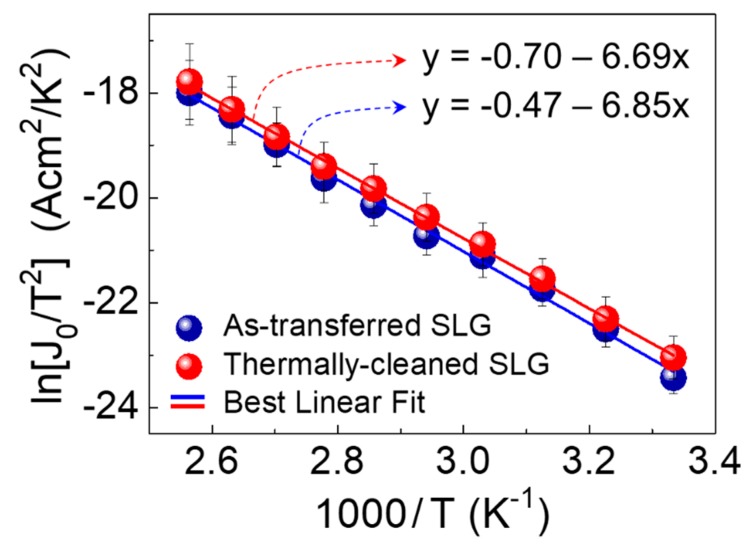
Richardson plots (i.e., ln(*J*_0_/T^2^) vs. 1000/T) for the ZnO/AT-SLG and the ZnO/TC-SLG Schottky PDs. The symbols are the experimental data points, and the solid lines are the best-fitted curves to determine A* for the PDs. The error bars indicate the standard deviation from data obtained from multiple samples fabricated by identical procedures (i.e., five devices of ZnO/AT-SLG Schottky PDs and five devices of ZnO/TC-SLG Schottky PDs, see also Supplementary Materials).

To characterize the Schottky barrier homogeneity of the ZnO/AT-SLG and ZnO/TC-SLG Schottky PDs, as a primary task, we measured the current–voltage (I–V) characteristics for both samples at temperatures ranging from 300 to 400 K in a dark chamber. For both samples, the diode current increased with increasing temperature (Figure 3a,b). Particularly, the increase in the reverse saturation current became significant because of thermally-activated carrier conduction (TACC) at elevated temperatures (see also the inset of Figure 3a). According to the thermionic emission theory [32], the I–V relationship of the Schottky diode at V > 3 kT/q can be expressed as:

(1) $$J=J_{0}\exp\left(qV/\eta kT\right)$$

(2) $$J_{0}={\mathrm{AA}}^{*}T^{2}\exp\left(-q\phi_{B}/kT\right),$$

where *J*_0_ is the reverse saturation current, *q* is the electron charge, *η* is the ideality factor, *k* is the Boltzmann constant, T is the absolute temperature, A is the contact area, and A* is the Richardson constant. Based upon Equations (1) and (2), we represented the Richardson plots (i.e., ln(*J*_0_/T^2^) vs. 1000/T) to determine the effective value of A* for our devices (Figure 4). From linear best fitting, the values of A* were calculated to be 0.495 and 0.628 A·cm^−2^K^−2^ for the ZnO/AT-SLG and the ZnO/TC-SLG Schottky PDs, respectively. The obtained A* values are much smaller than the theoretical value of the ZnO Schottky diode (≈32 A·cm^−2^K^−2^) because of the Schottky barrier inhomogeneity [33,34,35]. In addition, the ultrathin insulating layer at the Schottky interface could be a possible scenario that may degrade the A* value because the chemical adsorbates and oxygen molecules on SLG could locally form inadvertent insulating potential barriers [36,37,38]. Although both samples showed a smaller value of A* than the theoretical calculation, the effective value of A* was higher for the ZnO/TC-SLG Schottky PD (i.e., A* = 0.628 A·cm^−2^K^−2^) than the ZnO/AT-SLG Schottky PD (i.e., A* = 0.495 A·cm^−2^K^−2^). From this result, one can conjecture that the Schottky barrier homogeneity was enhanced in the ZnO/TC-SLG Schottky PD because the thermally-cleaned SLG exhibited a red-shift of G and 2D peaks, which is attributed to the effective elimination of oxygen molecules and/or chemical residues via thermal annealing at 280 °C (see also Figure 2b).

**Figure 5:**
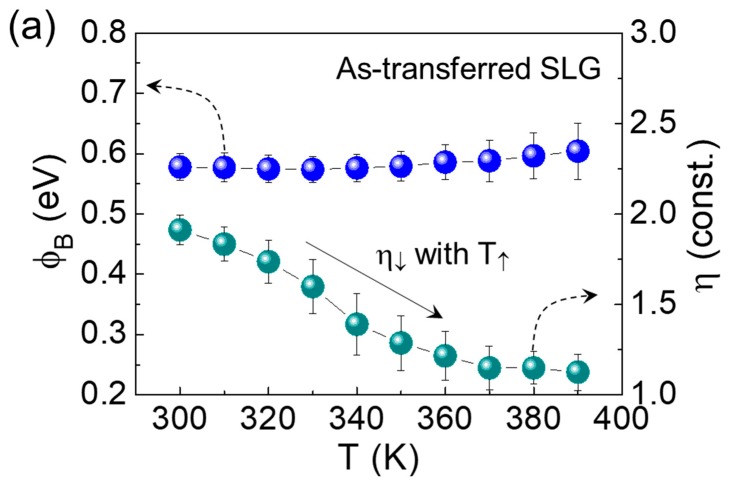

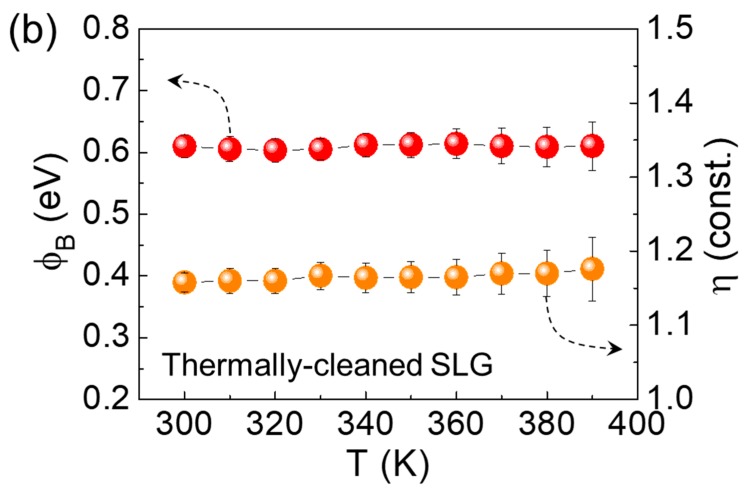
Magnitudes of *ϕ*_B_ and *η* as a function of the temperature for the (**a**) ZnO/AT-SLG and the (**b**) ZnO/TC-SLG Schottky PDs. The error bars indicate the standard deviation from data obtained from multiple samples fabricated by identical procedures (i.e., five devices of ZnO/AT-SLG Schottky PDs and five devices of ZnO/TC-SLG Schottky PDs, see also Supplementary Materials).

**Figure 6:**
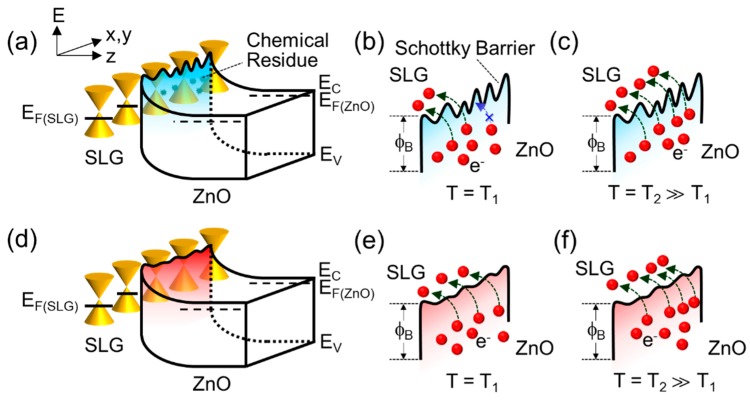
Expected Schottky barrier homogeneity along the in-plane direction normal to the surface and its impact on the carrier transport characteristics at different temperatures: (**a**) three-dimensional illustration of the energy-band diagram for the ZnO/SLG Schottky PD consisting of inhomogeneous Schottky barriers (e.g., for the case of the ZnO/AT-SLG Schottky PD), (**b**) carrier transport across the inhomogeneous Schottky barriers at T = T_1_, (**c**) carrier transport with TACC at T = T_2_ >> T_1_ for the diode with inhomogeneous Schottky barriers, (**d**) three-dimensional illustration of the energy-band diagram for the ZnO/SLG Schottky PD with homogeneous Schottky barriers (e.g., for the case of the ZnO/TC-SLG Schottky PD), (**e**) carrier transport across the homogeneous Schottky barriers at T = T_1_, and (**f**) carrier transport with TACC at T = T_2_ >> T_1_ for the case of the diode with homogeneous Schottky barriers.

To examine further insight into the Schottky barrier homogeneity, we investigated the temperature dependencies of *ϕ*_B_ and *η* for both samples. At 300 K, the ZnO/AT-SLG Schottky PD revealed a *ϕ*_B_ of 0.58 eV and *η* of 1.91 (Figure 5a). As we explained earlier, the Schottky barrier with *ϕ*_B_ = 0.58 eV was effectively formed due to the difference between Φ_SLG_ and χ_ZnO_. The high magnitude of *η* (= 1.91) is thought to be responsible for multiple recombination channels [39], which might arise from chemical residues residing at the ZnO/SLG interface. As the temperature increased, *ϕ*_B_ slightly increased while *η* suddenly decreased. These behaviors (i.e., *ϕ_B_* ↗ and *η* ↓ with T ↗) are commonly observed in typical metal/semiconductor Schottky diodes, and are known to be attributed to the lateral Schottky barrier inhomogeneity [40,41]. We therefore expect our ZnO/AT-SLG Schottky PD to have inhomogeneous Schottky barriers with a significant *ϕ*_B_ fluctuation along the surface direction, particularly at impurity and/or defect sites attributing to chemical residues (see also Figure 6a). In such a device, the effective values of *ϕ_B_* and *η* would increase and decrease with increasing temperature, respectively, because TACC through higher Schottky barriers became serious at elevated temperatures (see also Figure 6b,c).

For the ZnO/TC-SLG Schottky PD, *ϕ*_B_ and *η* were determined to be 0.61 eV and 1.16, respectively, at 300 K. In Schottky diodes, the low *η* manifested a clean interface between metal and semiconductor for the device, resulting in a dominance of thermionic emission rather than recombination [42]. One possible reason could be an effective removal of chemical residues from the SLG surface through the thermal cleaning process. Furthermore, the magnitudes of both *ϕ_B_* and *η* were almost independent of temperature (Figure 5b), which is totally different from the thermodynamic behavior of the ZnO/AT-SLG Schottky PD. In a homogeneous Schottky barrier system, due to the weak fluctuation of *ϕ_B_* along the surface direction, thermionic emission dominates its carrier transport whereas TACC becomes insignificant at elevated temperatures (see also Figure 6d,f). As a consequence, an enhanced Schottky barrier homogeneity will result in the almost invariance of both *ϕ_B_* and *η* upon varying the environmental temperature. Therefore, we expected that the Schottky barrier homogeneity was enhanced in our ZnO/TC-SLG Schottky PD via thermal cleaning (i.e., formation of uniform *ϕ_B_* along the surface direction by the effective removal of the chemical residues from the SLG surface).

**Figure 7:**
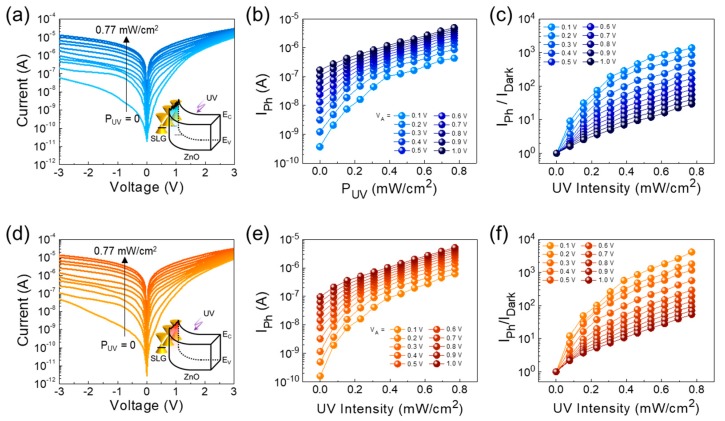
Comparison of the photoresponse characteristics between the ZnO/AT-SLG and the ZnO/TC-SLG Schottky PDs: I–V characteristics under 365-nm UV light illumination for the (**a**) ZnO/AT-SLG and the (**d**) ZnO/TC-SLG Schottky PDs, I_Ph_ as a function of P_UV_ for the (**b**) ZnO/AT-SLG and the (**e**) ZnO/TC-SLG Schottky PDs, and I_Ph_/I_Dark_ ratio as a function of P_UV_ for the (**c**) ZnO/AT-SLG and the (**f**) ZnO/TC-SLG Schottky PDs.

In Schottky PDs, the Schottky barrier homogeneity is one of the most crucial factors that strongly affects the photoresponse characteristics. To confirm the effect of the lateral homogeneity of the Schottky barrier, we assessed and compared the photoresponse properties of the ZnO/AT-SLG and ZnO/TC-SLG Schottky PDs under UV light illumination with a P_UV_ of 0–0.77 mW/cm^2^. For the ZnO/AT-SLG Schottky PD, as shown in Figure 7a, the current exponentially increased with increasing P_UV_ even in both the positive and the negative voltage regions. Figure 7b,c shows the P_UV_ dependence of the steady state photocurrent (i.e., I_Ph_ = I_Light_ − I_Dark_) at various bias voltages and its corresponding I_Ph_/I_Dark_ ratio, respectively. The device exhibited a large difference between I_Light_ and I_Dark_, resulting in a high I_Ph_/I_Dark_ ratio. Since the current level of I_Dark_ was sufficiently low at V = 0.1 V, the high I_Ph_/I_Dark_ ratio of ≈1100 was achievable at 0.1 V.

When illuminating the UV light onto the ZnO/TC-SLG Schottky PD, a similar behavior to the above was observed (Figure 7d). As can be seen from Figure 7e, however, the device exhibited a slightly lower current level of I_Dark_ (= 0.12 nA) at 0.1 V, compared to the ZnO/AT-SLG Schottky PD (I_Dark_ = 0.35 nA). We ascribe this feature to the lower contribution of carrier recombination at the small bias voltage in our ZnO/TC-SLG Schottky PD because the device revealed a quite low magnitude of *η* (= 1.16). Accordingly, the I_Ph_/I_Dark_ ratio was increased by a factor of ≈4 (i.e., I_Ph_/I_Dark_ ≈ 4200) at 0.1 V (Figure 7f), compared to the ZnO/AT-SLG Schottky PD. The low operating voltage was advantageous in both demonstrating a high on/off ratio and reducing the power consumption [43]; hence, we believe the ZnO/SLG Schottky structure is preferable for the application of low power UV photodetectors.

**Figure 8:**
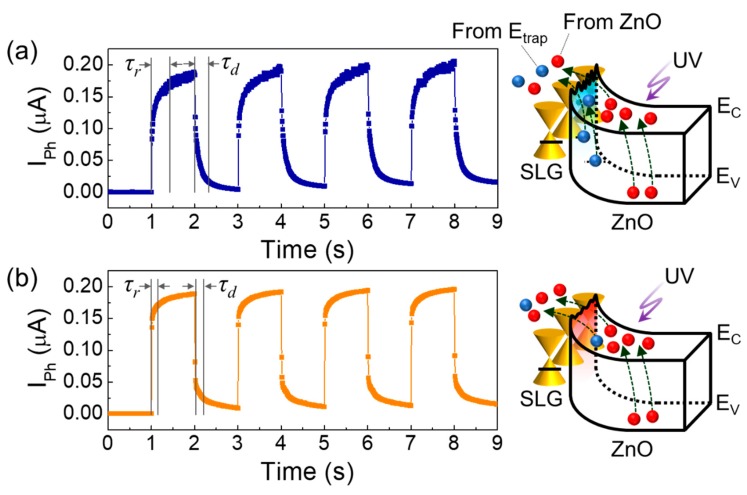
Photoresponse transient characteristics for the (**a**) ZnO/AT-SLG and the (**b**) ZnO/TC-SLG Schottky PDs under UV light illumination with P_UV_ = 0.77 mW/cm^2^. τ_r_ and τ_d_ were determined when the photoresponse had leached 90% and 10% of the on-state current time, respectively. The right-hand-side insets display the contribution of various photogenerated carriers to I_Ph_ under UV light illumination (blue dots: photocarriers from defect sites at the interface between ZnO/SLG, red dots: photocarriers from ZnO).

Figure 8 shows the transient waveforms of I_Ph_ for the ZnO/AT-SLG and ZnO/TC-SLG Schottky PDs under UV light illumination (P_UV_ = 0.77 mW/cm^2^) at an optical switching frequency of 0.5 Hz. Both the ZnO/AT-SLG and the ZnO/TC-SLG Schottky PDs exhibited a distinct, stable, and repeatable switching characteristic of I_Ph_ upon turning on and off the UV signal. However, the on-state photocurrent was unstable in the ZnO/AT-SLG Schottky PD (Figure 8a), whereas the ZnO/TC-SLG Schottky PD revealed a stable photoresponse of I_Ph_ at the UV on state (Figure 8b). The rising time (τ_r_) and the decay time (τ_d_) of the ZnO/TC-SLG Schottky PD were τ_r_ = 0.16 s and τ_d_ = 0.19 s, respectively; and those of the ZnO/AT-SLG Schottky PD were τ_r_ = 0.41 s and τ_d_ = 0.31 s, respectively. When considering the on- and off-state resistance (R_PD_ = 0.2–500 MΩ) and the electrostatic capacitance (C_PD_ ≈ 320 pF) of our devices, the time constant (i.e., τ_RC_ = R_PD_C_PD_) was determined to be 64 μs–160 ms. Therefore, the observed photoresponse time was close to the maximum τ_RC_ and is comparable to other graphene/ZnO Schottky PDs [11,12,13,14,15,16,44] (see also Table 1). For further improvement of the photoresponse time, a study on defect natures in ZnO can be the next step, for example, engineering of point defects and their lifetimes.

Here, one should focus on the fact that the photoresponse time of the ZnO/TC-SLG Schottky PD was outstandingly shorter than those of the ZnO/AT-SLG Schottky PD. We attribute such a discrepancy to the difference in Schottky barrier homogeneity between two devices. In the case of the inhomogeneous Schottky-barrier system (e.g., ZnO/AT-SLG Schottky PD), as illustrated in the right-hand-side inset of Figure 8a, the photocarriers would irregularly jump over the Schottky barrier due to the *ϕ_B_* fluctuation along the surface direction. In addition, some of photocarriers might suffer from charge-trapping at the ZnO/SLG interface due to the presence of residual chemical constituents and/or oxygen molecules. These cause the unstable on-state photocurrent and retard the photoresponse time. Contrariwise, such behaviors could be effectively diminished in the homogeneous Schottky barrier system (e.g., ZnO/TC-SLG Schottky PD) because the Schottky barrier undulation was suppressed via elimination of trap sites through thermal cleaning of the SLG surface (see the right-hand-side inset of Figure 8b). Owing to the enhanced Schottky barrier homogeneity, more stable and prompter photoresponse characteristics could be achievable from the ZnO/TC-SLG Schottky PD.

Finally, we discuss the responsivity (*R*) and the gain (*G*) of the prepared ZnO/SLG Schottky PDs. In PDs, *R* is defined by the ratio of the photocurrent to the optical power of incident light (P_opt_), and *G* is given by the ratio of the photogenerated carriers (N_e_) to the incident photons (N_Ph_). These can be described using following equations [45]:

(3) $$R=\frac{I_{\mathrm{Ph}}}{P_{\mathrm{opt}}}=\frac{I_{\mathrm{Light}}-I_{\mathrm{Dark}}}{P_{\mathrm{opt}}},$$

(4) $$G=\frac{N_{e}}{N_{\mathrm{Ph}}}=\frac{I_{\mathrm{Ph}}}{P_{\mathrm{opt}}}\times \frac{h\upsilon }{q},$$

where *hν* is the photon energy of the incident light. Using Equations (3) and (4), we calculated *R* and *G* for both the ZnO/AT-SLG and the ZnO/TC-SLG Schottky PDs. We here note that the average I_Ph_ value during a single pulse duration was used for calculations of *R* and *G*. In the case of the ZnO/AT-SLG Schottky PD, *R* and *G* were calculated to be ≈101 A/W and ≈347, respectively, and these are comparable to those of the state-of-the-art graphene/ZnO nanorod Schottky PDs [12,15] (see also Table 1). Compared to the ZnO/AT-SLG Schottky PD, both *R* and *G* were increased by ≈10% for the ZnO/TC-SLG Schottky PD (i.e., *R* ≈ 111 A/W and *G* ≈ 381). We attribute the increased *R* and *G* values to the enhanced Schottky barrier homogeneity in the ZnO/TC-SLG Schottky PD. In photocarrier conduction, *G* can also be defined as the ratio of the photocarrier lifetime (τ_PC_) in the photocarrier generation region to the carrier transit time (τ_TR_) in the photocarrier collection region [3]:

(5) $$G\approx \frac{\tau_{\mathrm{PC}}}{\tau_{\mathrm{TR}}}.$$

From this relation, we can expect τ_PC_ at the SLG/ZnO interface to be increased in the ZnO/TC-SLG Schottky PD, when assuming that τ_TR_ is identical in SLG. This specifies that the photocarrier diffusion length (*L*_PC_) is increased in the ZnO/TC-SLG Schottky PD because of the following relationship [32]:

(6) $$L_{\mathrm{PC}}=\sqrt{D_{\mathrm{PC}}\cdot \tau_{\mathrm{PC}}}$$

where *D*_PC_ is the diffusion coefficient of the photocarrier. In other words, *L*_PC_ is increased due to the decreased interface trap density resulting from the effective removal of the chemical residues and/or oxygen molecules at the SLG/ZnO interface via thermal cleaning of the SLG surface prior to the deposition of ZnO onto SLG.

## 4. Summary and Conclusions

We fabricated high-performance, low-power ZnO/SLG Schottky UV PDs by sputtering ZnO onto thermally-cleaned SLG sheets. The device clearly showed a rectifying behavior and exhibited an excellent photoresponse under UV illumination. When the UV light (P_UV_ = 0.62 mW/cm^2^) was irradiated onto the device, the I_Ph_/I_Dark_ ratio was recorded to be more than 4 × 10^3^. For the transient characteristics, the device represented a fast and stable photoresponse (i.e., τ_r_ = 0.16 s and τ_d_ = 0.19 s) upon switching of the UV light pulse. From analyses of the temperature-dependent I–V characteristics, we found such an outstanding photoresponse performance to arise from the enhanced Schottky barrier homogeneity due to the reduced interfacial trap density (i.e., effective removal of chemical residues and/or oxygen molecules at the ZnO/SLG interface via thermal cleaning).

## Figures and Tables

**Table 1:** Comparison of the photoresponse characteristics for various types of graphene/ZnO-based Schottky PDs.

Materials and Structures		λ_UV_ (nm)	V_B_ (V)	τ_r_ (s)	τ_d_ (s)	G	R	I_Ph_/I_Dark_	Ref.
SLG/ZnO TF	AT	365	0.1	0.41	0.31	≈347	≈101	1100	This Work
	TC		0.1	0.16	0.19	≈381	≈111	4200	
MLG/ZnO NRs		365	−5	0.7 × 10^−3^	3.6 × 10^−3^	385	113	<100	[12]
SLG/AlZnO NRs		380	SP	37 × 10^−6^	330 × 10^−6^	-	0.039		[11]
SLG/ZnO NRs		325	−1	0.3	0.5	-	21.26	≈30	[13]
SLG/ZnO NRs		325	−2	0.52	3.4		1.92	4	[14]
rGO/ZnO NRs		N/A	1	2.42	0.2	≈72	≈2.0	>>10^3^	[15]
rGO/ZnO NRs		365	SP	0.1	0.2			5	[16]
GQDs/ZnO NRs		340	−1						[44]

Note: TF, thin film; NRs, nanorods; SP, self-powered; GQDs, graphene quantum dots; **λ**_UV_, wavelength of UV light; V_B_, bias voltage.

## Supplementary Materials

The following are available online at https://www.mdpi.com/2079-4991/9/5/799/s1, Figure S1: I–V characteristic curves of various ZnO/AT-SLG Schottky PDs fabricated through an identical device fabrication process using as-transferred SLG, Figure S2: I–V characteristic curves of various ZnO/TC-SLG Schottky PDs fabricated through an identical device fabrication process using thermally-cleaned SLG.
