# Supplementary material for: Low-Power Graphene/ZnO Schottky UV Photodiodes with Enhanced Lateral Schottky Barrier Homogeneity
Source: Nanomaterials (Basel). 2019 May 24;9(5):799. doi: 10.3390/nano9050799 (PMC6566797; doi:10.3390/nano9050799)
Supplement: Supplementary file 1 [file nanomaterials-09-00799-s001.pdf]

Supplementary Materials

Low-Power Graphene/ZnO Schottky UV Photodiodes with Enhanced Lateral Schottky Barrier Homogeneity

Youngmin Lee [1], Deuk Young Kim [1,2] and Sejoon Lee [1,2,*]

[1] Quantum-Functional Semiconductor Research Center, Dongguk University - Seoul, Seoul 04623, Korea; ymlee@dongguk.edu (Y.L.); dykim@dongguk.edu (D.Y.K.)

[2] Department of Semiconductor Science, Dongguk University - Seoul, Seoul 04623, Korea

* Correspondence: sejoon@dongguk.edu; Tel.: +82-2-2260-3946; Fax: +82-2-2260-3945

■ Current–Voltage Characteristics of Various ZnO/AT-SLG and ZnO/TC-SLG Schottky PDs

When we fabricated the graphene/ZnO Schottky photodiodes (PDs) by direct sputtering ZnO onto single-layer graphene (SLG). Statistically, more than 85% of the graphene/ZnO Schottky PDs devices showed a clear rectifying behavior in their current–voltage (I–V) characteristics at room temperature. In Figures S1 and S2, we show multiple samples of graphene/ZnO Schottky PDs that have been fabricated through an identical device fabrication process using as-transferred (AT) SLG and thermally-cleaned (TC) SLG, respectively. Both the ZnO/AT-SLG Schottky PDs and the ZnO/TC-SLG Schottky PDs clearly revealed the obvious rectifying characteristics. The Schottky barrier height (ϕ_B) and the ideality factor (η) of the ZnO/AT-SLG Schottky PDs were 0.58–0.59 eV and 1.92–1.99, respectively, and were 0.60–0.61 eV and 1.17–1.21, respectively, for the ZnO/TC-SLG Schottky PDs.

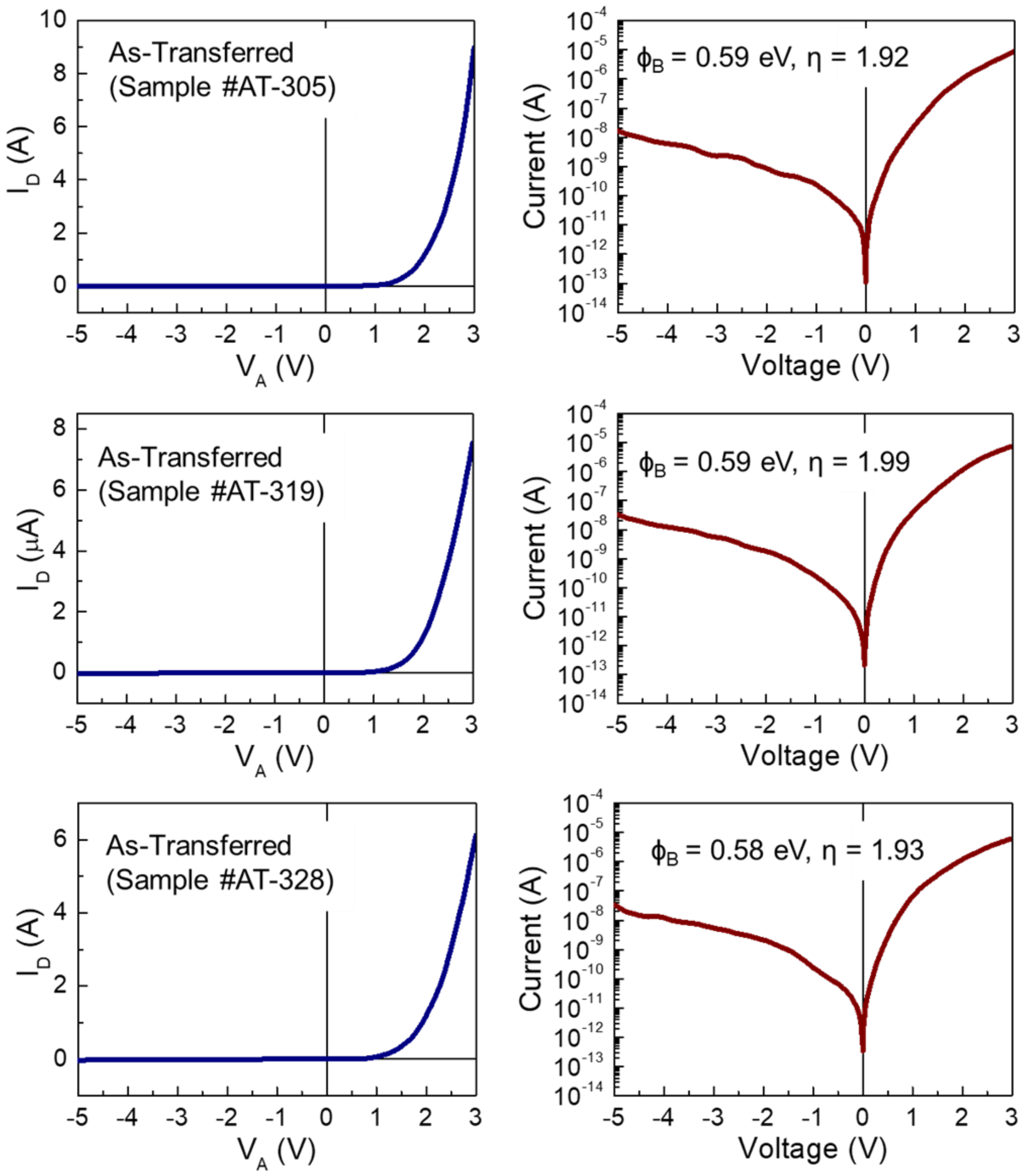

Figure S1. I–V characteristic curves of various ZnO/AT-SLG Schottky PDs fabricated through an identical device fabrication process using as-transferred SLG.

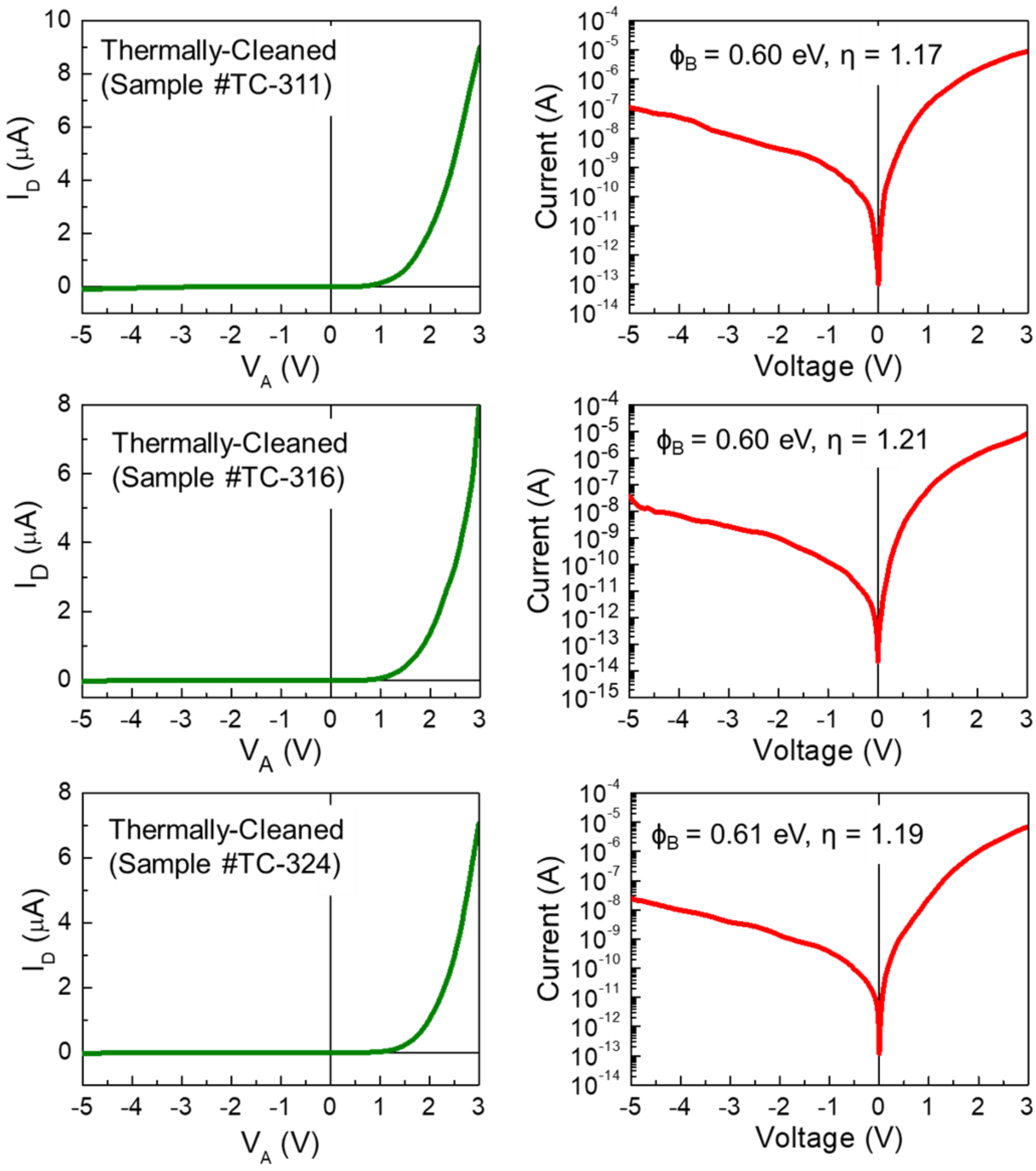

Figure S2. I–V characteristic curves of various ZnO/TC-SLG Schottky PDs fabricated through an identical device fabrication process using thermally-cleaned SLG.
